# Study on biodegradation kinetics of di-2-ethylhexyl phthalate by newly isolated halotolerant *Ochrobactrum anthropi* strain L1-W

**DOI:** 10.1186/s13104-020-05096-0

**Published:** 2020-05-24

**Authors:** Jean Bosco Nshimiyimana, Sujan Khadka, Piao Zou, Sanjib Adhikari, Ram Proshad, Alina Thapa, Li Xiong

**Affiliations:** 1 Department of Biochemistry and Molecular Biology, School of Life Sciences, Central China Normal University, Wuhan, 430079 China; 2 Department of Natural Resources and Environment Management, Protestant Institute of Arts and Social Science, Po Box 619, Huye, Rwanda; 3 Department of Microbiology, Birendra Multiple Campus, Tribhuvan University, Bharatpur, Chitwan, 44200 Nepal; 4 State Key Laboratory of Environmental Aquatic Chemistry, Research Center for Eco-Environmental Sciences, Chinese Academy of Sciences, Beijing, 100085 China; 5 Key Laboratory of Mountain Surface Process and Ecological Regulation, Institute of Mountain Hazards and Environment, Chinese Academy of Sciences, Chengdu, 610041 China; 6 State Key Laboratory of Alpine Ecology and Biodiversity, Institute of Tibetan Plateau Research, Chinese Academy of Sciences, Beijing, 100101 China; 7 University of Chinese Academy of Sciences, Beijing, 100049 China

**Keywords:** DEHP, Phthalates, Pollution, *Ochrobactrum anthropi* strain L1-W, Bioremediation

## Abstract

**Objective:**

Di-2-ethylhexyl phthalate (DEHP) pollution is one of the major environmental concerns all over the world. This research aimed at studying the biodegradation kinetics of DEHP by a newly isolated bacterial strain. Water and sediment samples were collected from Wuhan South Lake and potent bacterial isolates were screened for DEHP degradation, characterized by biochemical, physiological, morphological and *16S* rDNA gene sequencing, and optimized under suitable pH, temperature, NaCl and DEHP concentrations. DEHP and its metabolites were quantified by High Performance Liquid Chromatography and their degradation kinetics were studied.

**Results:**

The newly isolated bacterium was identified as *Ochrobactrum anthropi* strain L1-W with 99.63% similarity to *Ochrobactrum anthropi* ATCC 49188. It was capable of utilizing DEHP as the carbon source. The optimum growth temperature, pH, DEHP and NaCl concentration for the strain L1-W were 30 °C, 6, 400 mg/L and 10 g/L respectively. Strain L1-W was capable of degrading almost all (98.7%) of DEHP when the initial concentration was 200 mg/L within a period of 72 h. Besides, it was also found capable of degrading five other phthalates, thus making it a possible candidate for bioremediation of phthalates in the environmental settings.

## Introduction

Phthalates, also called as phthalate esters (PAEs), are chemical compounds belonging to the family of esters of phthalic acids. They are commonly used as plasticizers in polyvinyl chloride (PVC) and other polymers to enhance the durability as well as the elasticity [1]. Phthalates are the major components of wall coverings, food packagings, curtains, pesticides, rainwear, medical tubings, shoes and blood storage bags [2]. PAEs are less chemically bound in these products; consequently, they are likely to leach into the environment and contaminate water, air, soil or sediments [3].

PAEs are regarded as endocrine-disrupting chemicals (EDCs) [4], and are known to alter sexual differentiation [5]. DEHP is the most commonly used phthalate, and it has been classified among hazardous chemicals by the China National Environmental Monitoring Center, the European Community and the United States Environmental Protection Agency [6]. DEHP and its major intermediate end products-MEHP (mono(2-ethylhexyl) phthalate) and PA (phthalic acid)-have been found to impair with the respiratory and nervous immune system as well as development in humans [7].

Both hydrolysis and photolysis have been found to be ineffective to remove DEHP from the contaminated environment [8, 9], leaving biological degradation as the sole effective and reliable option to remove DEHP from both terrestrial and aquatic contaminated environments and restoring their natural conditions [10]. Microbial degradation has more advantages over hydrolysis and photolysis because it is cost-effective, faster and environment-friendly [11].

Previous studies have reported different microorganisms such as *Achromobacter denitrificans* [12], *Pseudomonas fluorescens* [13], *Bacillus megaterium* YJB3 [14], *Gordonia alkanivorans* YC-RL2 [15], *Mycobacterium* sp. NK0301 [16], *Providencia* sp. 2D [9] capable of degrading DEHP and its metabolites. In China, most of the DEHP degraders have been isolated from rivers, lakes, sediments, activated sludge, plastic recycling plants, compost amended soil and wetland [17, 18]. Only a few studies have documented successful biodegradation of DEHP with the use of microbes, although there are a lot of data available on the biodegradation of phthalates. In this context, a new and efficient bacterial strain was isolated, screened, characterized and optimized to study the biodegradation kinetics of DEHP.

## Main text

### Study design and setting

The work was conducted for 5 months from January to June 2019. The water and sediment samples were collected from Wuhan South Lake (Nanhu) in sterile plastic bottles and bags respectively and immediately brought to the Biochemistry and Molecular Biology unit laboratory of Central China Normal University, Wuhan, China, and stored in the refrigerator at 4 °C for further analysis.

### Methodology

Few days following the sample collection, 5 g of sediment and 5 mL of water samples were mixed and diluted with 45 mL distilled water (pH 7) in a small conical flask and left standing still on the bench overnight. Next, 5 mL of supernatant was inoculated into a 50 mL Luria–Bertani (LB) medium, thoroughly mixed with a magnetic stirrer and incubated in a rotary shaker adjusted at 28 °C and 180 rpm overnight. The broth culture was again sub-cultivated in inorganic salt agar (ISA) embedded with 500 mg/L of DEHP and incubated for 5–7 days. Only the bigger colony of bacteria capable of growing on ISA containing 500 mg/L of DEHP was selected for further studies. The physiological, biochemical and morphological characterization of the isolate was performed according to the Bergey’s Bacterial Identification Manual [19] and the Common Bacterial Identification Manual [20]. Extraction of bacterial genomic DNA was carried out by using the kit (Biospin Bacteria Genomic DNA Extraction kit). Later, PCR was performed using universal primers: forward primer 27F (5ʹ-AGAGTTTGATCCTGGCTCAG-3ʹ) and reverse primer 1492R (5ʹ-ACGGCTACCTTGTTACGACT-3ʹ) and gel electrophoresis was used to confirm the presence of DNA. The *16S* rDNA gene sequencing of the resulting products was done by Nanjing Bioheng Biotech Co., Ltd. Phylogenetic tree was constructed by using the neighbour-joining, maximum-parsimony and maximum-likelihood methods within the MEGA 7 software [21], and bootstrap values were calculated from 1000 replications.

In order to determine the optimal conditions of the isolate to degrade DEHP, single-factor optimization experiments were conducted with the following values: pH (4–8), temperature (15–45 °C) and NaCl concentrations (10–100 g/L). The growth of the isolate was evaluated by measuring OD_600_ (optical density at 600 nm) using UV-VIS spectrophotometer (Hitachi Industrial Components & Equipment, Singapore). The isolate was subjected to different concentrations of DEHP (maximum: 100–600 mg/L and minimum: 1–10 mg/L). After 72 h of incubation, the degradation ability was evaluated by measuring OD_600_. An uninoculated medium containing DEHP was used as a control in all the cases. The isolate was also tested for its ability to degrade wider ranges of PAEs. DEHP and its metabolites were analyzed in 12 h interval by using HPLC (Shimadzu Corporation, Kyoto, Japan) as previously described by Ren et al. [22]. The degradation kinetics were studied using first-order kinetics equation. The half-life of the DEHP with different initial concentrations was also measured.

### Results

#### Isolation and identification of DEHP degrading bacterium

**Fig. 1:**
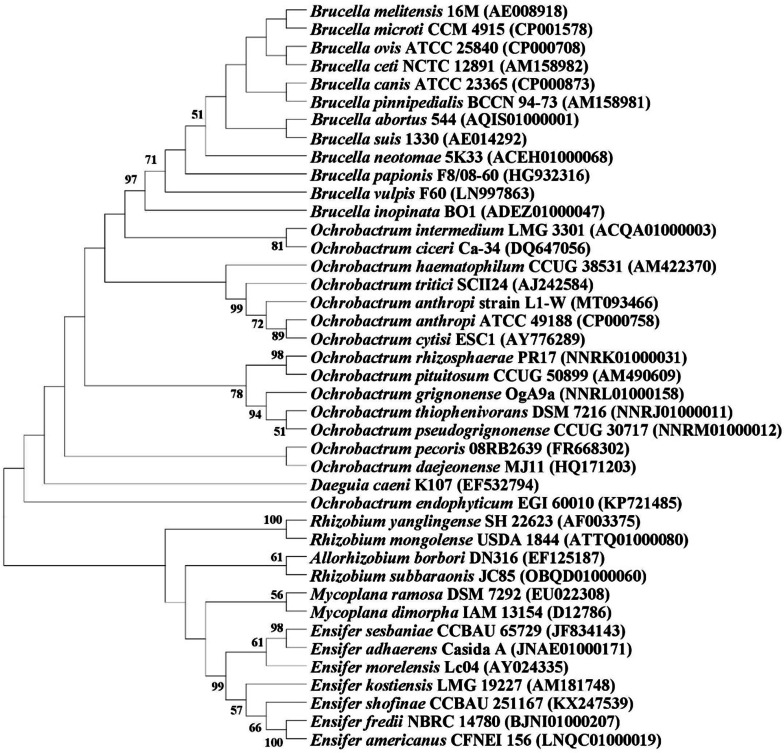
Phylogenetic tree based on *16S* rDNA gene sequences showing the position of *Ochrobactrum anthropi* strain L1-W. Bootstrap values (expressed as percentages of 1000 replications) of above 50% are shown at the branch points. The tree was reconstructed by using the neighbour-joining method

Morphological, biochemical and physiological characteristics of the isolate are mentioned in Additional file 1: Table S1. Phylogenetic tree revealed that the bacterium was 99.63% similar to *Ochrobactrum anthropi* ATCC 49188 (Fig. 1). Based on physiological, morphological, biochemical and *16S* rDNA gene sequence analysis, the isolate was identified as *Ochrobactrum anthropi* strain L1-W with gene bank accession number MT093466.

#### Effect of pH and temperature on the growth of strain L1-W

The pH dependence of strain L1-W with its growth was studied within the pH range of 4–8. The growth rate sharply increased with increase in pH up to 6. However, it started to decrease when the pH was beyond 6, suggesting that the optimum growth pH for the strain L1-W is 6 (Additional file 1: Figure S1). Similarly, the optimal temperature for biodegradation of DEHP was found to be 30 °C above which the growth rate declined along with the increase in temperature (Additional file 1: Figure S2).

#### Effect of DEHP and NaCl concentration on the growth of strain L1-W

Strain L1-W exhibited the highest growth rate when the DEHP concentration was 400 mg/L but decreased thereafter (Additional file 1: Figure S3). Likewise, strain L1-W withstood the salinity up to 100 g/L of NaCl exhibiting its halotolerant nature (Additional file 1: Figure S4).

#### Broad-spectrum substrate utilization

It was found that the strain L1-W can degrade at least six different PAEs; which makes it a plausible candidate for remediation of highly polluted environments (Additional file 1: Table S1).

#### Biodegradation kinetics of DEHP

Degradation kinetics of DEHP was assumed to follow the first-order reaction with respect to the following equation:

$$\ln \, C = \, - Kt \, + \, A$$

where *C, K, t* and *A* represent the concentration of DEHP, the first-order rate constant, time and a constant value respectively. The half-life of degradation of DEHP can be calculated by the formula: *t*_*1⁄2*_= *ln2/K* where *t*_*1/2*_ represents half-life.

**Fig. 2:**
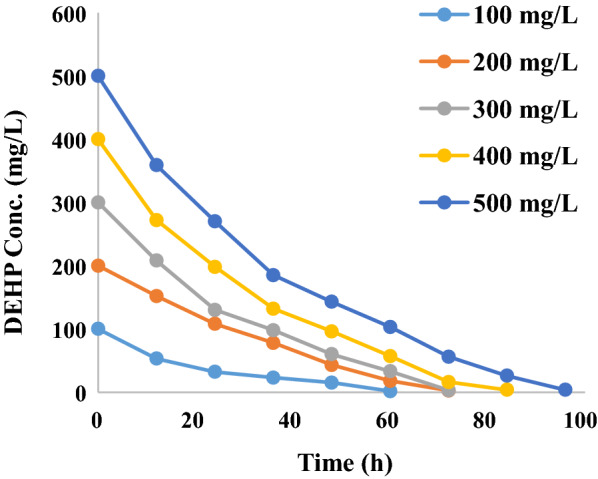
Degradation kinetics of DEHP by strain L1-W at various initial concentrations

Table 1 indicates kinetic equations of degradation of DEHP at various initial concentrations. It was found that 98.7% of DEHP was degraded in 72 h when the initial concentration was 200 mg/L with the half-life of 10.21 h (Fig. 2).

**Table 1:** Degradation kinetics of DEHP

Initial concentration (mg/L)	Kinetic equation	Half-life (h)
100	*ln C*= − 0.08995*t* + 3.57816	6.91
200	*ln C*= − 0.06119*t* + 4.21163	10.21
300	*ln C*= − 0.05845*t* + 4.62491	11.26
400	*ln C* = − 0.04112*t* + 5.13567	15.33
500	*ln C* = − 0.03826*t* + 5.32104	17.12

### Discussion

Wu et al. [23] reported the biodegradation of DBP by *Ochrobactrum* sp. which are regarded as one of the most effective microorganisms to survive the environments polluted by plasticizers because their cell wall and membrane are highly adapted to the harsh environment. High salinity content can affect the metabolic processes of the living organisms; therefore, reducing the ability to degrade DEHP [24]. Despite this, in the current study, strain L1-W showed a high salinity tolerance up to 100 g/L which suggests that it may reduce the cost of desalinization in solid and wastewater treatment. Many halotolerant phthalates degraders have been isolated from marine environments [25, 26]. Yang et al. [25] isolated strain *Rhodococcus ruber* YC-YT1 from saline water which could tolerate up to 120 g/L of NaCl. A study conducted by Jin et al. [27] has identified halotolerant isolate-*Sphingobium* sp.-which degraded DBP at the salinity ranging from 0 to 4%.

Studies have revealed that DEHP biodegradation is a temperature and pH-dependent process [10]. This is because biodegradation is usually carried out by multiple enzymes which work under specific pH and temperature [15]. A certain microorganism will be regarded as effective if it has the ability to degrade pollutants in a wide range of temperature and pH values. For the most DEHP-degraders, for example, *Acinetobacter* sp. LMB-5 [28], *Rhodococcus* WJ4 [29] and *Arthrobacter* sp. C21 [30], maximum degradation occurred at pH 7; however, they were unable to carry on degradation at higher or lower pH. On the other hand, some other microorganisms such as *Rhodococcus* sp. HS-D2 [31], *Gordonia alkanivorans* YC-RL2 [15], *Acinetobacter* sp. SN13 [11] and *Pseudomonas fluorescens* FS1 [13] have shown a wide pH range at 5–10, 6–11, 3–9, and 4–9 respectively. Compared with other degraders reported in various studies, the strain L1-W was capable of degrading DEHP in a wide pH range (4–8) and temperatures (15–45 °C), with the optimum degradation occurring at pH 6 and temperature 30 °C respectively. This is almost similar to the findings of Xiao-Hua et al. [32] who reported a similar optimum pH and temperature for the degradation of dichlorvos by *Ochrobactrum* sp., but unlike the current study, there was almost no activity of the degraders when the pH was less than 5.

It has been documented that more than one phthalate can exist simultaneously with other contaminants such as polychlorinated biphenyls (PCBs) in the same environment [33]. Several microorganisms have been reported capable of removing many PAEs from the environment. Wu et al. [23] have reported *Ochrobactrum* sp. capable of metabolizing three kinds of PAEs (DMP, DEP and DBP). It is worthy to note that not all bacteria have the ability to metabolize wider ranges of PAEs. For example, the research conducted by Sarkar et al. [34] concluded that *Gordonia* sp. Dop5 could not use PA. Compared with the above degraders, strain L1-W was found to be able to use at least six PAEs as its sole source of carbon, hence making it one of the excellent DEHP degraders with a wide range of substrate utilization ability.

Removing DEHP from its contaminated environment at low concentration raises some difficulties because if the concentration of the contaminant is very low it will hinder both growth and gene expression of the bacteria [10, 22]. On the other hand, a higher concentration of DEHP also inhibits the growth of bacteria [35]. In the current study, strain L1-W remained viable and active at the lowest and highest concentration of DEHP at 0.5 mg/L and 600 mg/L respectively. A study conducted by Yang et al. [25] reported that a strain *Rhodococcus ruber* YC-YT1 could survive and remain active at 0.5 mg/L and 1000 mg/L concentrations of DEHP. Nahurira et. al. [15] revealed that *Gordonia alkanivorans* strain YC-RL2 could not continue to degrade DEHP when the concentration was above 1000 mg/L. *Acinetobacter* sp. SN13 isolated by Xu et al. [11] showed the highest degradation of DEHP when the initial concentration of DEHP was 400 mg/L and it stopped when the concentration was above its optimum (500–1000 mg/L).

In the current study, degradation kinetics have revealed that the strain L1-W could degrade 98.7% of DEHP within 72 h when the initial concentration was 200 mg/L. Yang et al. [25] isolated *Rhodococcus rubber* strain YC-YT1 that could degrade 60% of DEHP after 3 days given that the initial concentration was 0.5 mg/L. Xu et al. [11] isolated *Acinetobacter* sp. SN13 and Nahurira et. al. [15] isolated *Gordoniaalkanivorans* strain YC-RL2 capable of removing more than 90% of DEHP within 5 and 7 days respectively. This showed that strain L1-W can degrade DEHP more efficiently compared to the other bacteria previously studied.

### Conclusions

*Ochrobactrum anthropi* strain L1-W, a newly isolated bacterial strain from the heavily polluted South Lake was characterized by physiological, morphological, biochemical and molecular techniques. Degradation of DEHP by the strain L1-W varied depending on the wide ranges of pH and temperature, and also with various concentrations of DEHP and NaCl. The broad substrate utilization tests revealed that other PAEs such as BBP, DMP, DEP, DBP and DBEP could also be easily degraded by the strain L1-W. Such capabilities make it a promising candidate for bioremediation process in the PAEs contaminated sites.

## Limitations

The study was unable to determine the formation of different intermediates and their routes under enzymatic actions exhibited by the strain L1-W. Several genes responsible for the biodegradation of DEHP were not studied.

## Supplementary information

**Additional file 1.** Additional figures and table.

## Data Availability

All datasets generated or analyzed during this study are included in the manuscript.

## Abbreviations

DEHP: Di-2-ethylhexyl phthalate
PAEs: Phthalate esters
PVC: Polyvinyl chloride
EDCs: Endocrine-disrupting chemicals
DMP: Di-methyl phthalate
DEP: Di-ethyl phthalate
DBP: Di-butyl phthalate
DBEP: Dibutoxy ethyl phthalate
DBP: Dibutyl phthalate
LB: Luria–Bertani
MEHP: Mono(2-ethylhexyl) phthalate
PA: Phthalic acid
PCBs: Polychlorinated biphenyls
