# Supplementary material for: Study on biodegradation kinetics of di-2-ethylhexyl phthalate by newly isolated halotolerant Ochrobactrum anthropi strain L1-W
Source: BMC Res Notes. 2020 May 24;13:252. doi: 10.1186/s13104-020-05096-0 (PMC7247211; doi:10.1186/s13104-020-05096-0)
Supplement: Supplementary file 1 — Additional file 1. Additional figures and table. [file 13104_2020_5096_MOESM1_ESM.docx]

**Supplementary Figure 1 Effect of pH on the growth of strain L1-W**

**Supplementary Figure 2** **Effect of temperature on the growth of strain L1-W**

**Supplementary Figure 3 Effect of DEHP concentration on the growth of strain L1-W**

**Supplementary Figure 4 Effect of NaCl concentration on the growth of strain L1-W**

**Supplementary Table 1** **Morphological, physiological, biochemical and substrate utilization characteristics of strain L1-W**

| **Attributes** | **Characteristics** |
| --- | --- |
| Gram’s reaction | Gram-negative |
| Bacterial shape | Small rods |
| Colony characteristics | Irregular, moist and flat |
| **Biochemical tests** | |
| Maltose | Positive |
| Lactose | Positive |
| Glucose | Positive |
| Mannose | Positive |
| Galactoside | Positive |
| Simon Citrate | Negative |
| Catalase | Positive |
| Oxidase | Positive |
| Motility | Motile |
| H_2_S production | Positive |
| **Substrate utilization tests** | |
| DMP | ++ |
| DEP | ++ |
| DBP | ++ |
| DBEP | + |
| BBP | + |

**++: Vigorous growth; +: Moderate growth**

**DMP: Dimethyl phthalate, DEP: Diethyl phthalate, DBP: Dibutyl phthalate, BBP: Benzyl butyl phthalate, DBEP: Dibutoxy ethyl phthalate**
